# Autogenous bone graft in the management of post-osteomyelitis bone defects in children in a limited-resource setting – a retrospective cohort study with a minimum follow-up of 7 years

**DOI:** 10.5194/jbji-10-155-2025

**Published:** 2025-04-15

**Authors:** Antonio Loro, Fulvio Franceschi, Muhumuza M. Fisha, Emmanuel Ewochu, Geoffrey Mwanje, Annamaria Dal Lago, Martin McNally

**Affiliations:** 1 Department of Orthopaedics, CoRSU Rehabilitation Hospital, Kisubi, Uganda; 2 independent researcher: Trento, Italy; 3 Department of Paediatrics, CoRSU Rehabilitation Hospital, Kisubi, Uganda; 4 Honorary Consultant in Limb Reconstruction, Oxford Bone Infection Unit, Oxford University Hospitals, Oxford, UK

## Abstract

**Background.** Post-osteomyelitis bone defects represent a challenging clinical situation. This retrospective cohort study was designed to evaluate the long-term outcome of the use of non-vascularized bone grafts in the management of such defects in children. **Methods.** Twenty-three children (mean age 7 years, range 2–13 years) were studied. All of the defects were segmental (mean defect length 6 cm, range 3–12 cm), involving the tibia, femur, humerus and radius. Fifteen children presented with an active infection and were managed with a staged protocol. The first stage included sequestrectomy or debridement of the site. The second stage, i.e. the graft procedure, was performed after 12 weeks on average. The mean follow-up was 9.2 years (range 7–15 years). **Results.** Bone union was primarily achieved in 14 children (61 %). Complications were experienced in the remaining nine children. Conservative and surgical treatment led to bone union in all patients within 5 years of the index procedure. Recurrence of infection was observed in two patients (8.7 %). All of the children were able to use the limb at the final follow-up; only three required the use of a brace. **Conclusions.** Autogenous non-vascularized bone graft may be considered a valid option in the treatment of bone defects secondary to osteomyelitis in children.

## 1 Introduction

Adequate debridement is the most important principle in the surgical management of paediatric osteomyelitis, particularly in the developing world, where bone infections are endemic and often seen in advanced stages. Surgery is often, but not always, required due to the chronicity of the disease at presentation (Loro et al., 2022). However, debridement may produce bone defects whose management can be challenging.

Bone reconstruction may be achieved in several ways, but all require consideration of clinical and logistical factors. Child age, defect size and location, soft tissue status and the available skills and resources of the surgical team must be considered (Bezstarosti et al., 2021; Loro et al., 2022). In addition, in some regions of the developing world, the socio-economic situation of the child's family must be evaluated properly because treatment may be resource-intensive.

Segmental bone reconstruction is complex and ideally should be performed in well-equipped centres. However, these centres may be located far from the isolated areas where most cases occur. Ongoing transportation costs may be impossible for families to sustain, resulting in poor clinic compliance.

Intercalary defects of the long bones (mainly the tibia, femur, humerus and radius) may be managed with conventional non-vascularized bone graft (NVBG) (Patwardham et al., 2013), bone transport (El-Rosasy, 2013; Loro et al., 2023), vascularized bone graft (Loro et al., 2021), Masquelet's technique (Canavese et al., 2017), the excision–graft technique according to Papineau (1974), conversion to a one-bone forearm (Peterson, 2008) and transference of the ipsilateral fibula (Fowles et al., 1979; Zahiri et al., 1997). Previous papers have reported our experience in bone transport (Loro et al., 2023) and vascularized fibula flap (Loro et al., 2021) in the management of bone defects in a paediatric population. The present study was designed to clarify whether grafts longer than 6 cm were in need of vascular supply as advised by the old “6 cm rule” (Weiland et al., 1983) or whether NVBG was a valid option even in larger defects in children as reported by Allsopp et al. (2016).

Our study included children operated on in two different centres: in the first 2 years (2007–2009), the surgeries were performed in another Ugandan hospital until CoRSU opened in 2009. CoRSU is a private, non-profit facility with the core mandate of offering orthopaedic and plastic surgery services to children, with a focus on the most disadvantaged and vulnerable. Disadvantaged children are defined as having one meal per day, walking barefoot, being tasked with fetching water or firewood and sharing a single room with parents and siblings. Vulnerable children have difficult access to education and health facilities, with their time often spent caring for siblings or domestic animals, thus being robbed of their right to play.

This study evaluated the clinical outcomes of conventional bone grafting in surgical management of paediatric osteomyelitis in two specialist centres in Uganda.

## 2 Methods

### 2.1 Study design

This retrospective cohort study evaluated the long-term bone and functional results of a staged reconstruction technique for bone defects secondary to haematogenous osteomyelitis in children. This study is reported using the STROBE guidelines for cohort studies; further details can be found in Table S1 in the Supplement.

In the period under review (2007–2015), 46 non-vascularized graft procedures were done, and 23 eligible children were included based on the following criteria:

- age below 14 years at surgery;
- presence of a bone defect following surgical debridement for osteomyelitis;
- no previous reconstruction attempts;
- reconstruction with an autogenous graft, either tibial cortex or fibula strut, inserted without internal fixation;
- stabilization achieved with mono-lateral external fixators; and
- a minimum follow-up of 7 years.

Of the 23 children that were excluded, 16 had a short follow-up and 7 were untraceable.

Twenty-two were traced and invited for a voluntary clinical and radiological examination. The data of one child who did not attend the last follow-up are included in the complication outcomes as they were available via medical records, but they have been excluded from the long-term outcomes.

The two primary outcomes were the bone and functional status at final follow-up. Bone status was assessed using five parameters: union, infection relapse, deformity, need for bracing and limb length discrepancy (LLD). Bone union was achieved if radiologically there were sound integration of the graft and continuity of three out of four cortices. Infection relapse was judged clinically by recurrence of sinus drainage, local features of inflammation, pyrexia and pain at the site of the previous infection; biochemical markers were not utilized. Functional status was based on pain, joint function, the ability to perform activities of daily living (ADLs) and the requirement of a walking aid. The ability to perform ADLs was a self-reported rating for activities, e.g. the ability to walk to and from school, fetch water (cans of 5, 10 or 20 L) or firewood, dig, wash clothes, cook and take care of younger siblings. Secondary outcomes recorded complications encountered during treatment, mainly graft infection, recurrence of infection and non-union.

### 2.2 Treatment protocol

#### 2.2.1 Stage 1

**Figure 1:**
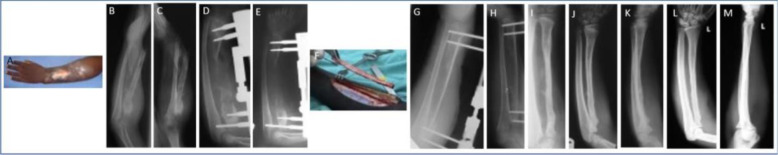
An 8-year-old boy with a 5-month history of untreated, spontaneous infection of the left radius. At presentation, the sequestrated radius was partly extruded **(a)**. Radiographs showed the sequestration of the entire diaphysis, with a large bone defect **(b, c)**. Reconstruction stage 1 required sequestrectomy (debridement) and positioning of a paediatric Orthofix rail **(d)**. After surgery, the bone ends were gradually distracted to restore the radial length and the distal radius–ulna joint relationship **(e)**. One month later, a long tibial strut was employed, tightly inserted between the bone ends and stabilized by a new external fixator **(f, g)**. Post-operative radiographs were taken 70 **(h)**, 120 **(i)** and 210 **(j, k)** days after stage 2. It took 7 months to achieve the cylindrical transformation of the strut, with excellent integration at both ends. Radiographs at 11 years after surgery showed full radial integration and remodelling **(l, m)**. Excellent bone and functional results were obtained.

**Figure 2:**
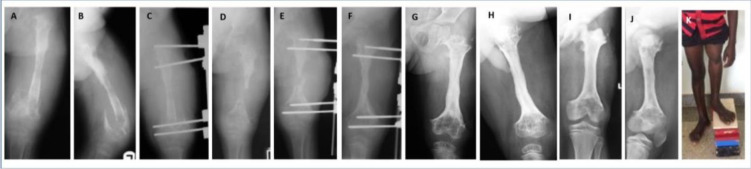
A 2-year-old child with a severe case of haematogenous femoral osteomyelitis presenting 2 months after the onset of symptoms. Radiographs showed sequestration of the entire diaphysis, pathological fracture and physeal involvement **(a, b)**. Debridement and stabilization with an external fixator were performed, leaving the sequestrum in situ **(c)**. This allowed better demarcation of the sequestrated segment and time for development of periosteal involucrum. This is common in small children. Sequestrectomy was performed 3 months later. Ten months later, the child presented with a bone defect in the mid-femur without any sign of infection **(d)**. A tibial cortical strut graft was placed and secured by trans-osseous stitches. An external fixator was reapplied **(e)**. Sound integration of the graft was observed at 16 weeks **(f)** and the fixator was removed. Radiographs are shown 1 year after the graft procedure **(g, h)**. Graft hypertrophy and cylindrical transformation of the graft were noted, together with early closure of both physes, deformity of the femoral condyles and patella baja. An orthoprosthesis for the leg length discrepancy (LLD) was fabricated. At 8 years, he was walking without aids or a shoe raise. He had a 17 cm LLD and no infection relapse. The radiographs showed a short femur, knee deformity and patella baja **(i, j, k)**.

The first stage of the reconstruction was the same for all of the children, i.e. removal of the sequestra or dead tissues and limb stabilization with external fixation (except in two femoral infections which were immobilized with hip spica) (Figs. 1, 2, 4 and 5). Debridement was strictly limited to removing the necrotic tissues, preserving the viable inflamed tissues which may contribute to healing. Intraoperative specimens were routinely collected for histopathology; tuberculous osteomyelitis was detected in a single case. Specimens for microbiology were only taken in two cases, both of them cultured *Staphylococcus aureus*. In six cases with exposed sequestra, mixed flora was assumed to be present. In the other 15 cases, 8 of which had quiescent infection, microbiological testing was not done.

Spacers and local antibiotics were not employed in dead-space management. A 7 d course of oral antibiotics, mainly semi-synthetic penicillin, was routinely prescribed post-operatively.

#### 2.2.2 Stage 2

**Figure 3:**
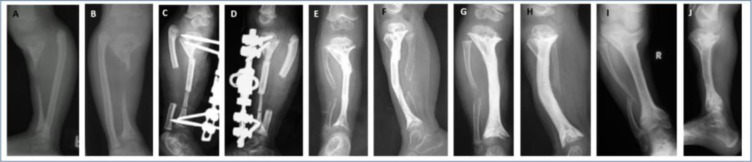
A 2-year-old girl presented with severe osteomyelitis and an exposed sequestrum. The infection was quiescent at presentation. Radiographs showed a short deformed leg, a large bone defect of the tibia and early physeal closure **(a, b)**. Amputation was advised but was refused. A segment of the ipsilateral fibula was inserted between the trimmed bone ends, with concurrent axis correction and external fixation **(c, d)**. At 13 weeks, the graft is integrating and remodelling **(e, f)**. Radiographs at 14 months showed a short, sclerotic tibia with moderate procurvatum **(g, h)**. An orthoprosthesis was prescribed. Ten years later, she had no infection relapse, just moderate knee and ankle pain with a severe LLD (15 cm). She walked normally without aids or a brace. The radiographs showed a short tibia with varus deformity due to medial tibial plateau depression **(i, j)**.

**Figure 4:**
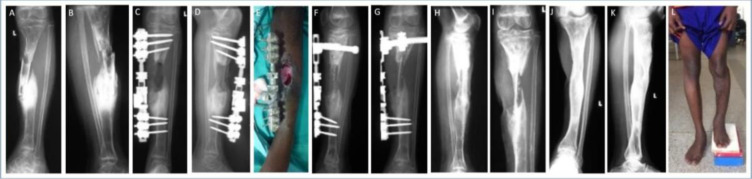
A 12-year-old boy presented with complicated tibial osteomyelitis with an exposed sequestrum, pathological fracture as well as bone and soft tissue loss. Radiographs showed sequestration of the central shaft of the tibia **(a, b)**. Sequestrectomy was performed, and the tibia was stabilized with an external fixator. A proximally based hemisoleus flap was transferred 2 weeks after the debridement **(c, d, e)**. Three months later, a tibial cortex strut was inserted **(f, g)**. The fixator was removed 6 weeks later and replaced with a long-leg cast. Radiographs are shown at 4 months after grafting **(h, i)**. Seven years later, they showed full graft integration, no infection recurrence and moderate varus of the ankle. **(j, k, l)** The patient reported moderate pain in the knee and ankle joints after long walks. The LLD was 7 cm, but the patient refused any shoe raise. Proposal for further surgery for lengthening was put on hold by the family.

The second stage involved autogenous bone grafting which was inserted when the soft tissue envelope was clinically normal and there were no local signs of infection (mean interval 12 weeks, range 2–34 weeks). Laboratory tests such as erythrocyte sedimentation rate (ESR) or C-reactive protein (CRP) were not used in the decision-making process. Tibial cortex strut was used in 19 patients and fibula strut in 4 patients. A hemisoleus flap was used in one case to close the soft tissues in the middle third of the leg. The bone graft was performed 2 months later when the flap had healed well (Fig. 4). The tibial strut, with its periosteum, was harvested from the upper third of the contralateral tibia (usually the medial cortex), while the fibula graft was taken from the same leg in three cases out of four. The ipsilateral non-vascularized fibular graft allowed the correction of the concurrent angular deformities in the same session (Fig. 3). Drill holes along the contour of the graft were united using a narrow, thin osteotome and the strut was gently removed.

Pre-graft distraction was used in all cases of radial loss to re-establish an acceptable distal radius–ulna joint relationship (Fig. 1). No distraction was used in the lower limb defects. This would have been technically difficult due to the short lengths of the bone ends and the inadequate bone quality for sound purchase of the pins (Figs. 2 and 3).

**Figure 5:**
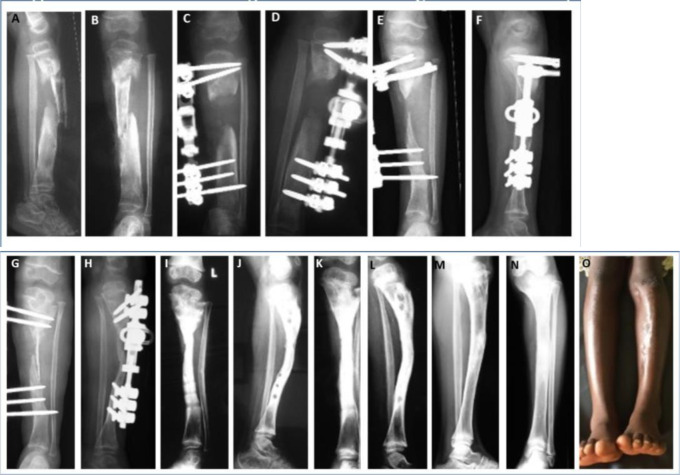
A 2-year-old girl presented 2 months after spontaneous onset of infection, with an exposed sequestrum, pathological fracture and loss of soft tissues **(a, b)**. Sequestrectomy and external fixation were carried out **(c, d)**, leaving the skin to heal by secondary intention. Pin loosening required a change in the frame, but two proximal screws were erroneously inserted into the upper physis **(e, f)**. Six months after the initial debridement, a tibial cortex strut was inserted and the fixator was replaced **(g, h)**. Radiographs are shown at 5 **(i, j)** and 7 **(k, l)** months of grafting. Sound graft incorporation and bone modelling may be observed. Seven years later, at the age of 12 years **(m, n, o)**. Closure of the upper tibial physis led to a LLD of 2 cm and deformity of the tibial plateau. However, good bone and functional results were achieved in the long term.

No internal fixation was employed. Cancellous bone chips were packed at both ends. Stabilization of the segment was always done with a mono-lateral external fixator, without the guidance of an image intensifier.

#### 2.2.3 Follow-up

Clinical and radiological follow-up was undertaken regularly in the first 2 years after surgery. After this, follow-up became infrequent for the majority of the children.

## 3 Results

### 3.1 Patient characteristics

Twenty-three children (12 males) were enrolled in the study. The mean age at presentation was 7 years (range 2–13). All the defects were segmental (mean length 6 cm, range 3–12 cm): 17 involved the lower limbs (13 tibial, 4 femoral) and 6 the upper limbs (3 humeral and 3 radial). In 12 of the children the defect was 6 cm or longer.

Fifteen of the children had active infections at presentation, and all had a complicated infection (Type 2) (Loro et al., 2022). Eight of the children were treated for osteomyelitis elsewhere and presented with a quiescent post-surgical defect. They were classified as Type 3 (Loro et al., 2022). All the children would have been classed as Cierny–Mader Type 4 (Cierny and Mader, 1984) or BACH complex (Hotchen et al., 2020). Table 1 summarizes the features of the children at presentation.

**Table 1:** Patient characteristics.

	n (%)
Males	12 (52)
Females	11 (48)
Mean age	7 years (range 2–13 years)
Bone defect	
Mean size	6 cm (range 3–12 cm)
Site	
Femur	4
Tibia	13
Humerus	3
Radius	3
Associated features at presentation	
Exposed sequestra with skin loss	6
Pathological fracture	15
Limb length discrepancy	11
Septic arthritis	3
Severe joint contracture and stiffness	9
Inability to use the affected limb	12
Active infection at the site	15
Use of a walking aid	10

Radiologically, three tibiae and two femora showed closure of both physes (Figs. 2 and 3), and the tibiae showed closure of a single physis. Two humeri had closure of the distal physis, and dislocation of the radial head was detected in one of them.

### 3.2 Complications

Primary union was uneventfully reached in 14 patients (61 % of the cases) by 1 year after surgery (mean incorporation time 16 weeks, range 11–24 weeks).

Complications in the remaining nine patients are highlighted in Table 2. The management was both conservative and surgical, achieving sound union in all 23 cases within 5 years of the index procedure.

One tuberculous humeral infection was followed by complete reabsorption of the tibial graft, and the child was successfully treated with a vascularized fibula flap 2 years later. The diagnosis of tuberculosis was unknown at the time of the graft, despite previous biopsies, and was discovered at the time of graft removal.

In another case, a fibular graft survived an early infection, but recurrence followed 1 year later. A successful debridement was followed by non-union. Union was achieved with several casts and braces. Tibial lengthening was performed 2 years later.

Another case of tibial non-union, 6 months from the index procedure, was treated conservatively with casts and braces. A further case of non-union was only stabilized and healed with external fixation, without opening the site. It took 2 months to heal. Recurrence of the infection occurred 2 years later: four surgeries were performed within 5 years to control the infection.

The remaining three cases of non-union required site debridement and external fixation. All three healed, but one suffered a late refracture (healed with conservative casting) and one had a recurrence of infection, which was still present at the last review. Additionally, two other patients had recurrence of infection 4 and 7 years from the index procedure. Debridement led to sound healing in both cases.

Finally, another patient had two procedures of tibial lengthening to correct LLD.

**Table 2:** Summary of the major outcomes of the series.

Outcome of index surgery	Number (%)	Secondary treatment	Final outcome
Uneventful healing (at 1 year)	14 (61)	None	Infection-free, healed
Early graft infection	1 (4.3)	Excision and freely vascularized fibular graft	Infection-free, healed
Early graft infection + non-union + recurrent infection	1 (4.3)	Debridement, bracing and secondary tibial lengthening	Infection-free, healed
Non-union	3 (13)	Two cases with debridement and external fixation One case with cast and bracing	All infection-free, healed
Non-union + recurrent infection	2 (8.7)	One case with external fixation and repeated debridement for infection One case with debridement and external fixation	Both cases healed One case with continued infection
Recurrent infection	2 (8.7)	One case with debridement One case with debridement and rotational flap	Both infection-free, healed

Technical complications were experienced, mainly in fixator pin positioning. Wrong screw positioning was observed in two cases (Fig. 3). Pins were inserted into the open physis (one tibia, one femur); this likely contributed to limb shortening and angular deformity.

In two cases requiring exchange of the screws for septic loosening, new pins were either wrongly positioned or had a poor purchase, and they were backed within a few weeks.

In one case, the fibular graft was harvested too distally without stabilizing the lateral malleolus. This led to a proximal migration of the malleolus, followed by a valgus deformity of the ankle joint.

No problems were encountered in the healing process of the donor site.

### 3.3 Bone and functional results at the last follow-up

Twenty-two patients were followed to a mean of 9.2 years (range 7–15 years). Nineteen were students, one a homemaker, one s butcher and one a shopkeeper. The child with tuberculous infection of the humerus was lost to follow-up.

Bone continuity was restored in all of the patients; all but three were walking without assistive devices or using the upper limb without bracing. Recurrence of infection was observed in two patients (8.7 %). One patient, who experienced a post-graft valgus ankle deformity, raised complaints about the donor site. LLD was observed in 17 of the 22 patients, and 11 out of 23 had LLD prior to treatment. Eleven patients had a discrepancy between 2 and 5 cm, and six patients had a discrepancy between 6 and 19 cm. Three patients with 10, 18 and 19 cm discrepancies were wearing an orthosis. The remaining patients denied the need for shoe raise in their daily life.

Radiologically, all of the grafts underwent hypertrophy. In addition, the tibial cortex underwent geometrical transformation remodelling into a cylindrical shape and restored the continuity of the medullary canal (Figs. 1, 2 and 4).

At the final follow-up, 15 of the patients reported no pain in the operated segment, 5 complained of mild pain and 2 complained of moderate pain (mostly associated with long walks or carrying heavy objects).

Eleven patients had normal range of motion in the adjacent joints. Four had stiff knees, three stiff ankles and two stiff wrists. One patient had a fused ankle, one a fused elbow and one a fused knee. However, severe restriction of the joint motion had already been noted at presentation.

When asked about the ability to carry out ADLs, 15 patients reported no difficulty, 3 had mild difficulty, 2 had moderate difficulty and 2 had severe difficulty in performance.

## 4 Discussion

NVBG is an accepted method of treatment in the management of infected segmental defects. These are usually employed in a staged protocol (McNally et al., 1993). The use of cancellous grafts, iliac crest grafts and tibial and fibula struts has been reported in defect reconstruction in both upper (Rasool, 2011) and lower (Rasool, 2008) limbs. In our unit, these various options are still routinely used in the management of post-osteomyelitic defects in children.

Focal bone loss, particularly in the tibia, can be treated with the technique of open cancellous grafting described by Papineau (1974). His original three-stage technique was modified, mainly around the number of debridements before grafting, the time of grafting itself and the management of the soft tissues (Emami et al., 1995; Panda et al., 1998). We have reserved it for a few cavitary, resistant defects located in the distal half of the tibia.

Our experience is also very limited with iliac crest grafting, a technique reported in the literature (Panda et al., 1998; Daoud and Saighi-Bouaouina, 1989). Big defects in small children, as described, require a large quantity of graft that may not be possible to harvest. Donor site complications, such as persistent pain or nerve damage, must also be considered (Ebraheim et al., 2001).

Prior to the development of orthoplastic techniques, our preferred method of treatment was the one described in this paper, even for large segmental defects. The method was found to not be technically difficult, and assembly of complex external frames was not required. This was a good option when orthoplastic facilities were not available. The largest defects included in this series would probably have been treated with different techniques if microvascular surgery had been available. Technically speaking, this method does not require super-specialized centres or skills, since the graft harvesting and insertion can be done in any hospital with a functioning surgical unit. However, the surgeon does require good skills in debridement, good external fixation and suitable equipment. Pin positioning may be difficult in small bones, especially if the remaining bone ends are short. Without the guidance of the C arm, iatrogenic damage to the physes is a possibility.

In developing countries, regular follow-up is still only partially achieved, often due to socio-economic challenges (Baldan et al., 2014). In osteo-articular infections, children tend to be lost to follow-up once the infection becomes quiescent and adequate mobility is regained. As a consequence, few data are reported on outcomes of specific treatments. After initial treatment, many of the children did not attend regular follow-up, but we were able to trace all but one child. It took 1 year to trace the children enrolled in this study, mostly by volunteers who first gathered information and then physically visited the villages. Using social media might be possible in other settings but was not practicable for our cohort, considering issues such as lack of electricity, Internet cost and availability as well as civil unrest or war in the country of residence. In this study, we were able to review 22 out of 23 (96 %) children for a minimum of 7 years, providing good evidence of the final outcome and the need for further surgery after the index procedure.

At the final follow-up, all of the children had bone union with sound graft integration and bone modelling, irrespective of the length of the defect. This impressive remodelling, which is expected in children, produced good bone alignment, even in the few cases where the initial strut positioning was not perfect. Bad alignment was not related to the development of non-union. A role in the complex process of bone modelling could be ascribed, hypothetically, to the periosteum, which was routinely harvested with the tibial cortex or fibular shaft.

Our study confirmed the report by Allsopp et al. (2016) that the graft can be successful even in defects exceeding 6 cm, thereby avoiding the need for microvascular anastomosis. It was surprising to observe that this was also obtained in the few children who had a clear clinical and radiological picture of non-union at the graft–host junction. In the long term, full recovery was achieved just through normal load bearing, without the use of bracing.

The method was successful in avoiding infection relapse, with only two cases (8.7 %) needing re-debridement several years after the procedure. One infection was, however, far from the graft site and could be related to migration to bone of untreated pin site sepsis.

The complications experienced were those commonly reported in the literature, i.e. infection, non-union and fracture (Daoud and Saighi-Bouaouina, 1989; Ebraheim et al., 2001). They were unrelated to the size of the defect. Numerically speaking, they were less numerous when this method was compared to vascularized grafts or bone transport (Loro et al., 2021, 2023). All were successfully managed by additional surgeries but had serious repercussions for the family, with increased treatment costs, hospital stays and compliance with the management plan.

We showed that it is not essential to fix the graft with internal fixation in children; it is important to use a few trans-osseous stitches or, in the case of the fibular strut, insert the graft tightly between the bone ends. This could lower the risk of graft infection since the use of plates or wires is unnecessary. Stabilization with an external fixator is the recommended method, and this has to be properly maintained, with regular review, until integration is achieved. It could be argued that poor stability might have been one of the co-factors behind some of the cases of non-union. However, it is not clear that additional internal fixation would have prevented these.

Good soft tissue cover is mandatory for effective healing, so the graft should be inserted once local conditions suggest that infection is eradicated. Depending on this, the time gap between debridement and graft may be variable, ranging from a few weeks to months (Daoud and Saighi-Bouaouina, 1989). This is particularly important with advanced cases of osteomyelitis, since extruded sequestra and soft tissue loss are common at presentation.

Bone reconstruction requires long treatment, which must be explained clearly to the child's relatives or caregivers. The surgeon must honestly explain that, due to the severity of the infection, limitation of limb function and limb length discrepancy are real possibilities, affecting over half of our patients. The same honesty must be applied when explaining that a considerable cost will be incurred by multiple surgeries, follow-ups, dressings and transportation.

In our series, some children had infections which would be considered for amputation and prosthetic fitting. This option was always refused by the family and clan. It is a treatment that should be discussed, particularly with major LLD or limited function, but the availability of future prostheses must be considered in this decision.

This study is limited by its size, which could introduce some bias into the results. However, there are very few published series on this technique, particularly in children, and usually with only a few cases. Out of all the eligible children, only one was excluded from long-term follow-up due to not being contactable, but we did include this child in the early outcomes with complications. The size of the study was limited by the number of children who were excluded due to short-term follow-up. However, this was considered a non-negotiable criterion by the authors, as long-term follow-up was a crucial endpoint.

We received children from a wide area around the hospital, including from neighbouring countries, so we believe that this cohort is representative of cases seen in our geographical region. The results will be generalizable to similar areas with limited healthcare resources and patients who often present late after the onset of infection. However, these results may not be applicable in other healthcare settings.

## 5 Conclusion

This study demonstrates the validity of the technique in the treatment of post-infectious bone defects in children. A defect exceeding 5–6 cm is not a contraindication. Good bone reconstruction and a low rate of infection relapse can be expected in the long term. The procedure can be carried out in appropriately equipped surgical centres. As with other reconstruction techniques, strict patient supervision is one of the keys to success.

## Supplement

10.5194/jbji-10-155-2025-supplement The supplement related to this article is available online at https://doi.org/10.5194/jbji-10-155-2025-supplement.

## Supplement

10.5194/jbji-10-155-2025-supplement
10.5194/jbji-10-155-2025-supplement The supplement related to this article is available online at https://doi.org/10.5194/jbji-10-155-2025-supplement.

## Data Availability

The data that support the findings of this study are openly available from figshare at https://doi.org/10.6084/m9.figshare.28689080 (Loro and Loro, 2025).

## Appendix

The supplement related to this article is available online at https://doi.org/10.5194/jbji-10-155-2025-supplement.
