# Supplementary material for: Autogenous bone graft in the management of post-osteomyelitis bone defects in children in a limited-resource setting – a retrospective cohort study with a minimum follow-up of 7 years
Source: J Bone Jt Infect. 2025 Apr 15;10(2):155–63. doi: 10.5194/jbji-10-155-2025 (PMC12082333; doi:10.5194/jbji-10-155-2025)
Supplement: The supplement related to this article is available online at https://doi.org/10.5194/jbji-10-155-2025-supplement. [file jbji-10-155-2025-supplement.pdf]

Supplement of J. Bone Joint Infect., 10, 155–163, 2025
https://doi.org/10.5194/jbji-10-155-2025-supplement
© Author(s) 2025. CC BY 4.0 License.

Supplement of

Autogenous bone graft in the management of post-osteomyelitis bone defects in children in a limited-resource setting – a retrospective cohort study with a minimum follow-up of 7 years

Antonio Loro et al.

Correspondence to: Antonio Loro (antonioloro@libero.it)

The copyright of individual parts of the supplement might differ from the article licence.

Table S1: STROBE Statement—checklist of items that should be included in reports of observational studies

	Item No.	Recommendation	Page No.	Relevant text from manuscript
**Title and abstract**	1	(*a*) Indicate the study's design with a commonly used term in the title or the abstract	1	Title: A retrospective cohort study with a minimum follow-up of seven years.
		(*b*) Provide in the abstract an informative and balanced summary of what was done and what was found	2	Abstract
**Introduction**				
Background/rationale	2	Explain the scientific background and rationale for the investigation being reported	3	
Objectives	3	State specific objectives, including any prespecified hypotheses	3	This retrospective study evaluated the long-term bone and functional results of a staged reconstruction technique for bone defects secondary to haematogenous osteomyelitis in children.
**Methods**				
Study design	4	Present key elements of study design early in the paper	3-4	Methods section
Setting	5	Describe the setting, locations, and relevant dates, including periods of recruitment, exposure, follow-up, and data collection	3-4	Introduction and Methods section with description of hospital, patient inclusions and follow-up.
Participants	6	(*a*) *Cohort study*—Give the eligibility criteria, and the sources and methods of selection of participants. Describe methods of follow-up<br>*Case-control study*—Give the eligibility criteria, and the sources and methods of case ascertainment and control selection. Give the rationale for the choice of cases and controls<br>*Cross-sectional study*—Give the eligibility criteria, and the sources and methods of selection of participants	4	Eligibility described under inclusion criteria. Follow-up described, page 7.

		(*b*) *Cohort study*—For matched studies, give matching criteria and number of exposed and unexposed<br>*Case-control study*—For matched studies, give matching criteria and the number of controls per case	NA	
Variables	7	Clearly define all outcomes, exposures, predictors, potential confounders, and effect modifiers. Give diagnostic criteria, if applicable	4	Section 2.1 Methods
Data sources/ measurement	8*	For each variable of interest, give sources of data and details of methods of assessment (measurement). Describe comparability of assessment methods if there is more than one group	4	Section 2.1<br>No comparable group
Bias	9	Describe any efforts to address potential sources of bias	13	Limitation section
Study size	10	Explain how the study size was arrived at	3	All children who had the inclusion criteria in the study period, at our institution and who could be traced for follow-up.

Continued on next page

Quantitative variables	11	Explain how quantitative variables were handled in the analyses. If applicable, describe which groupings were chosen and why	NA	Descriptive cohort only
Statistical methods	12	(*a*) Describe all statistical methods, including those used to control for confounding	NA	
		(*b*) Describe any methods used to examine subgroups and interactions	NA	
		(*c*) Explain how missing data were addressed	NA	
		(*d*) *Cohort study*—If applicable, explain how loss to follow-up was addressed<br>*Case-control study*—If applicable, explain how matching of cases and controls was addressed<br>*Cross-sectional study*—If applicable, describe analytical methods taking account of sampling strategy	NA	
		(*e*) Describe any sensitivity analyses	NA	
**Results**				
Participants	13*	(a) Report numbers of individuals at each stage of study—eg numbers potentially eligible, examined for eligibility, confirmed eligible, included in the study, completing follow-up, and analysed	3, 4	Methods section
		(b) Give reasons for non-participation at each stage	3-4	Single patient description
		(c) Consider use of a flow diagram	NA	
Descriptive data	14*	(a) Give characteristics of study participants (eg demographic, clinical, social) and information on exposures and potential confounders	8	Table 1
		(b) Indicate number of participants with missing data for each variable of interest	NA	
		(c) *Cohort study*—Summarise follow-up time (eg, average and total amount)	10	22 patients with mean follow-up of 9.2 years (range 7-15).
Outcome data	15*	*Cohort study*—Report numbers of outcome events or summary measures over time	9,10	Table 2
		Case-control study—Report numbers in each exposure category, or summary measures of exposure	*NA*	
		Cross-sectional study—Report numbers of outcome events or summary measures	*NA*	
Main results	16	(*a*) Give unadjusted estimates and, if applicable, confounder-adjusted estimates and their precision (eg, 95% confidence interval). Make clear which confounders were adjusted for and why they were included	NA	
		(*b*) Report category boundaries when continuous variables were categorized	NA	
		(*c*) If relevant, consider translating estimates of relative risk into absolute risk for a meaningful time period	NA	

Other analyses	17	Report other analyses done—eg analyses of subgroups and interactions, and sensitivity analyses	NA	
**Discussion**				
Key results	18	Summarise key results with reference to study objectives	12	
Limitations	19	Discuss limitations of the study, taking into account sources of potential bias or imprecision. Discuss both direction and magnitude of any potential bias	13	New Limitations section
Interpretation	20	Give a cautious overall interpretation of results considering objectives, limitations, multiplicity of analyses, results from similar studies, and other relevant evidence	13	Conclusion
Generalisability	21	Discuss the generalisability (external validity) of the study results	13	Referred to in new limitations
**Other information**				
Funding	22	Give the source of funding and the role of the funders for the present study and, if applicable, for the original study on which the present article is based	14	New statement of funding

*Give information separately for cases and controls in case-control studies and, if applicable, for exposed and unexposed groups in cohort and cross-sectional studies.

Note: An Explanation and Elaboration article discusses each checklist item and gives methodological background and published examples of transparent reporting. The STROBE checklist is best used in conjunction with this article (freely available on the Web sites of PLoS Medicine at http://www.plosmedicine.org/, Annals of Internal Medicine at http://www.annals.org/, and Epidemiology at http://www.epidem.com/). Information on the STROBE Initiative is available at www.strobe-statement.org.
